# Fine mapping of the tomato yellow leaf curl virus resistance gene *Ty*-*2* on chromosome 11 of tomato

**DOI:** 10.1007/s11032-014-0072-9

**Published:** 2014-03-28

**Authors:** Xiaohui Yang, Myluska Caro, Samuel F. Hutton, John W. Scott, Yanmei Guo, Xiaoxuan Wang, Md Harunur Rashid, Dora Szinay, Hans de Jong, Richard G. F. Visser, Yuling Bai, Yongchen Du

**Affiliations:** 1 Institute of Vegetable and Flowers, The Chinese Academy of Agricultural Sciences, 12 Zhongguancun Nandajie, Haidian District, Beijing, 100081 China; 2 Shandong Key Laboratory for Biology of Greenhouse Vegetables, Institute of Vegetables and Flowers, Shandong Academy of Agricultural Sciences, Jinan, China; 3 Gulf Coast Research and Education Center, University of Florida, 14625 CR 672, Wimauma, FL 33598 USA; 4 Wageningen UR Plant Breeding, Wageningen University and Research Center, Droevendaalsesteeg 1, 6708 PB Wageningen, The Netherlands; 5 Laboratory of Genetics, Wageningen University, Droevendaalsesteeg 1, 6708 PB Wageningen, The Netherlands

**Keywords:** Breeding, Resistance, Tomato, Tomato yellow leaf curling virus (TYLCV), Virus-induced gene silencing

## Abstract

Resistances to begomoviruses, including bipartite tomato mottle virus and monopartite tomato yellow leaf curl virus (TYLCV), have been introgressed to cultivated tomato (*Solanum lycopersicum*) from wild tomato accessions. A major gene, *Ty*-*2* from *S. habrochaites* f. *glabratum* accession “B6013*,”* that confers resistance to TYLCV was previously mapped to a 19-cM region on the long arm of chromosome 11. In the present study, approximately 11,000 plants were screened and nearly 157 recombination events were identified between the flanking markers C2_At1g07960 (82.5 cM, physical distance 51.387 Mb) and T0302 (89 cM, 51.878 Mb). Molecular marker analysis of recombinants and TYLCV evaluation of progeny from these recombinants localized *Ty*-*2* to an approximately 300,000-bp interval between markers UP8 (51.344 Mb) and M1 (51.645 Mb). No recombinants were identified between TG36 and C2_At3g52090, a region of at least 115 kb, indicating severe recombination suppression in this region. Due to the small interval, fluorescence in situ hybridization analysis failed to clarify whether recombination suppression is caused by chromosomal rearrangements. Candidate genes predicted based on tomato genome annotation were analyzed by RT-PCR and virus-induced gene silencing. Results indicate that the NBS gene family present in the *Ty*-*2* region is likely not responsible for the *Ty*-*2*-conferred resistance and that two candidate genes might play a role in the *Ty*-*2*-conferred resistance. Several markers very tightly linked to the *Ty*-*2* locus are presented and useful for marker-assisted selection in breeding programs to introgress *Ty*-*2* for begomovirus resistance.

**Electronic supplementary material:**

The online version of this article (doi:10.1007/s11032-014-0072-9) contains supplementary material, which is available to authorized users.

## Introduction

Tomato-infecting begomoviruses, including the monopartite tomato yellow leaf curl virus (TYLCV) and numerous bipartite viruses, are transmitted by the adult sweet potato whitefly [*Bemisia tabaci* (Gennadius) biotype B], which is also known as the silverleaf whitefly (*B. argentifolii* Bellows and Perring). These viruses cause serious losses to tomato (*Solanum lycopersicum* L.) production in many tropical and subtropical regions in the world (Ji et al. 2007a; Cohen and Lapidot 2007). Whitefly control measures such as the use of insecticides and/or fine-mesh screens or UV-absorbing plastic films/screens can limit disease damage, but epidemics can still occur. Also, whitefly resistance to the used chemicals has been reported (Antignus et al. 2001; Horowitz et al. 2007). Thus, deployment of resistant cultivars offers an attractive method to control these diseases. Cultivated tomato is susceptible to TYLCV, so breeding efforts rely on the transfer of resistance genes from wild tomato relatives. Species that have demonstrated resistance include *S. pimpinellifolium, S. peruvianum, S. chilense, S. habrochaites*, and *S. cheesmaniae* (Ji et al. 2007b; Pico et al. 1996; Scott 2007; Vidavski 2007). So far, as many as five resistance loci have been mapped, i.e., the dominant genes including *Ty*-*1*, *Ty*-*2*, *Ty*-*3*, *Ty*-*4*, and recessive gene *ty*-*5* (Zamir et al. 1994; Hanson et al. 2000; Ji et al. 2007a, 2009b; Anbinder et al. 2009). *Ty*-*1* and *Ty*-*3* were each derived from *S. chilense* and mapped to nearby positions on chromosome 6 (Ji et al. 2007c); however, Verlaan et al. (2013) demonstrated that *Ty*-*1* and *Ty*-*3* are alleles of the same gene. *Ty*-*4*, also derived from *S. chilense*, was mapped to chromosome 3 (Ji et al. 2008). The recessively inherited *ty*-*5* gene, first identified in the breeding line TY172 and later found in material derived from “Tyking,” was mapped to chromosome 4 (Anbinder et al. 2009; Hutton et al. 2012). The *ty*-*5* gene is likely derived from a complex of *S. peruvianum* accessions (Anbinder et al. 2009). However, there is also evidence showing that *ty*-*5* is a loss-of-function mutation that likely occurred in cultivated tomato (Levin et al. 2013). *Ty*-*2* was derived from *S. habrochaites* f. *glabratum* accession “B6013” (Kalloo and Banerjee 1990; Ji et al. 2009a) and was previously mapped to the long arm of chromosome 11 near markers TG36 (84 cM) and TG393 (103 cM) (Hanson et al. 2000). Further research indicated that *Ty*-*2* was localized to an introgression spanning markers TG36 (84 cM) to TG26 (92 cM) (Hanson et al. 2006). Later, *Ty*-*2* was delimited to a shorter introgression spanned by markers C2_At1g07960 (82.5 cM) and T0302 (89 cM) (Ji et al. 2009a), a distance of at least 500,000 bp on the tomato genome assembly. The fusarium wilt race 2 resistance gene (*I*-2) is close to the *Ty*-*2* region (Simons et al. 1998), and there may be difficulty in combining these important resistances in *cis*. Reducing the *Ty*-*2* introgression would be helpful in combining these two important disease resistances in a single line (Ji et al. 2009a).

Because *Ty*-*1* and *Ty*-*2* are both dominant and provide high levels of resistance to many strains of TYLCV, they are widely utilized by breeders. Yet, neither gene is effective against bipartite begomoviruses, and the resistance of both has been overcome by some strains of TYLCV (Ji et al. 2007a). There is evidence, however, that *Ty*-*2* can provide an enhanced level of resistance to bipartite begomoviruses when pyramided with *Ty*-*3* (Mejía et al. 2005), potentially making it a more attractive tool to breeders. Very recently, the cloning of *Ty*-*1* and *Ty*-*3* showed that they code for a DFDGD-class RNA-dependent RNA polymerase (RDR) for which no clear function has yet been described. Also, in the same study, it was shown that *Ty*-*2* does not encode for a RDR (Verlaan et al. 2013). Thus, cloning additional genes for TYLCV resistance offers a unique opportunity to advance the insight into novel types of resistance genes. The objective of this research is to fine map *Ty*-*2* toward the cloning of the gene.

## Materials and methods

### Plant materials used in Florida, US

H9205 is an H.J. Heinz Inc., processing tomato hybrid with TYLCV resistance conferred by *Ty*-*2* in its heterozygous status. In a previous study by Ji et al. (2009a), F_2_ progeny from H9205 was screened for recombination and three F_2_ recombinants (i.e., 82, 108, and 134) were identified. One of these plants (No. 134) was heterozygous for *Ty*-*2* contained within an introgression from C2_At1g07960 (82.5 cM) to T0302 (89 cM). This plant was advanced to the F_3_ generation, and progeny heterozygous for the same region was self-pollinated to produce an F_4_ population used in the present study. In total, 11,000 individual F_4_ plants were screened in two phases for recombination within the *Ty*-*2* region.

For Phase I of the recombinant screening, 4,000 F_4_ progeny were screened in Fall 2009 and 30 plants were identified that contained crossover events between the markers C2_At1g07960 and T0302. Recombinants were categorized into two groups: Group A was composed of individuals carrying one chromosome with a recombined introgression and one chromosome with no introgression. Group B was composed of individuals carrying one recombined and one nonrecombined introgression. These plants were transplanted to the field in mid-October and allowed to self-pollinate, and seeds were harvested. In Spring 2010, progeny lines of Group A recombinants, along with resistant and susceptible controls, were inoculated and transplanted to small pots in the greenhouse. Plants were evaluated for disease severity, and from each resistant line, F_5_ seed was harvested from one or two plants that were homozygous for the recombined introgression. In Fall 2010, 24 seedlings from each of the Group B recombinants were grown in a greenhouse. Recombinant inbred lines (RILs) were developed by genotyping each plant and selecting individuals that were homozygous for the recombined introgression. Plants of each RIL, along with controls, were inoculated and transplanted to 3.8-L pots in the greenhouse for the evaluation of disease severity.

For Phase II testing, approximately 7,000 additional F_4_ plants were screened in Spring 2011 for recombination between markers C2_At1g07960 and T0302. Plants with recombination events between C2_At1g07960 and M1 were selected and transferred to the field in April 2011, and selfed seed was harvested from each plant. In Fall 2011, 48 seedlings from each recombinant were genotyped, and individuals homozygous for the recombined introgression were selected representing RILs for TYLCV inoculation and field evaluation of disease severity. RILs were evaluated in a randomized complete block design with two blocks and 4- to 5-plant plots.

In all inoculated experiments, “Horizon” was used as the susceptible control and an F_5_ breeding line homozygous for *Ty*-*2* was used as the resistant control. The *Ty*-*2* breeding line was developed from H9205 by self-pollinating to the F_5_ generation while selecting for homozygosity of the entire introgression originally present in the hybrid.

### Plant materials used in Wageningen, the Netherlands

One advanced breeding line and one F_2_ population, both derived from commercial hybrids harboring the *Ty*-*2* gene in the genetic background of *S. lycopersicum*, were provided by breeding companies within the cooperative framework of the Centre for BioSystems Genomics (CBSG). The F_2_ population was used for recombinant screening. The F_2_ plants selected from recombinant screenings were selfed, and their F_3_ progenies were used for further testing with TYLCV. The advanced breeding line was used for gene expression and virus-induced gene silencing experiments. For all the experiments, plants were grown under greenhouse conditions (23 °C, 60 % humidity, and 16-/8-h day/night cycle).

### Inoculation and disease evaluation

In Florida, United States, all plants tested were inoculated with whiteflies viruliferous for the Israeli strain of TYLCV and subsequently assessed for disease severity according to the method described by Griffiths and Scott (2001) with some modifications. Briefly, 4- to 6-week-old seedlings were exposed to viruliferous whiteflies for one to 2 weeks in a growth chamber. Following inoculation, the whiteflies were killed and the plants were transplanted to 3.8-L pots in the greenhouse or to the field. Plants were rated for TYLCV disease severity approximately 40 days after exposure to whiteflies. Plants without symptoms similar to the resistant control were rated R and plants with severe symptoms similar to the susceptible control were rated S. There were no intermediate reactions.

In Wageningen, the Netherlands, TYLCV infection was done using *Agrobacterium*-mediated inoculation using the infectious TYLCV-IL clone as previously described by Verlaan et al. (2011). In all disease tests, *S. lycopersicum* cv. Moneymaker (MM) was used as the susceptible control.

### Molecular markers

All markers used in this study were PCR-based, including sequence-characterized amplified region (SCAR) markers and cleaved amplified polymorphic sequence (CAPS) markers (Table S1). These were either publicly available or were designed from version SL2.40 of the tomato genome assembly by BatchPrimer3 online (http://probes.pw.usda.gov/batchprimer3/index.html).

### Quantitative RT-PCR

For gene expression analysis, leaf samples from the top part of three plants per genotype were taken at 0, 9, and 20 days after TYLCV inoculation at Wageningen (the Netherlands). Two genotypes were used, the Wageningen *Ty*-*2* line (see description above) and tomato cultivar *S. lycopersicum* cv. Moneymaker (MM). Total RNA was isolated from leaf tissue using Qiagen RNA easy Plant Mini Kit, according to the manufacturer’s protocol. cDNA was synthesized using the iScript cDNA Synthesis kit (Bio-Rad). Quantitative real-time RT-PCR was performed using a Bio-Rad iCycler iQ5, in a 10 μL reaction (employing SYBR Green Supermix) and according to the Bio-Rad protocol. Primers were designed to amplify a 100- to 200-bp region of each candidate gene from tomato *Ty*-*2* cDNA. Primer3 online software was used for primer selection, and conditions were settled following the recommendations of Thornton and Basu (2011). As a reference, the ubiquitin gene was used with primers UBI-F (5′-GGACGGACGTACTCTAGCTGAT-3′) and UBI-R (5′-AGCTTTCGACCTCAAGGGTA-3′).

### Virus-induced gene silencing (VIGS)

cDNA sequences of candidate genes predicted in the *Ty*-*2* region were obtained from the Sol Genomics Network database. Primers were designed to amplify a 150- to 450-bp region from cDNA of the Wageningen *Ty*-*2* line using Phusion DNA polymerase. Fragments targeting the candidate genes for silencing were amplified and cloned into pENTR-TOPO (Invitrogen), sequenced for confirmation and subsequently cloned into TRV2 vector (Liu et al. 2002) using the Gateway system. Plasmids were transformed into *Agrobacterium tumefasciens* strain GV3101. For sequence alignments, MEGA version 5 software was used.

Virus-induced gene silencing (VIGS) experiments were performed as described in Verlaan et al. (2013). Briefly, TRV infection was done through *Agrobacterium*-mediated infiltration on cotyledons of 10-day-old seedlings using syringes without needle. Two weeks after TRV inoculation, agro infiltration with TYLCV was performed.

### Fluorescent in situ hybridization (FISH) analysis

Slide preparations, BAC isolation, and FISH were carried out as described in Verlaan et al. (2011).

## Results

### *Ty*-*2* maps between UP8 and M1, a region of about 300 kb

Phase I screening of approximately 4,000 seedlings in Fall 2009 resulted in the identification of 30 plants having a recombination event between C2_At1g07960 and T0302 (Table 1). Progeny lines of Group A recombinants were phenotypically evaluated. Eight recombinants (A1 and A2) that were segregating for the upper portion (C2_At1g07960 to M1) of the *Ty*-*2* introgression also segregated for resistance, while the five recombinants (A3 and A4) that were lacking this upper portion of the introgression were all susceptible. Likewise, evaluation of Group B RILs in Fall 2010 confirmed that those containing the upper portion of the introgression were resistant (B1–B3, Table 1), while those lacking this region were susceptible (B4–B7, Table 1). Thus, the genotype and phenotype results of important recombinants clearly delimited *Ty*-*2* to the region below UP8 (data of A1–A2 and B1–B3) and above M1 (data of B3 and B4).

**Table 1:** Genotype for the UP8 to T0302 marker interval of *Ty*-*2* recombinants identified from Phase I screening and their phenotype as determined by testing their progenies

Group	No. of recombinants	Genotype							Spring 2010 progeny phenotype		Fall 2010 RIL phenotype	
		UP8	C2_At1g07960	C2_At3g52090	M1	M2	M3	T0302	Tested plants	Resistant plants	Tested plants	Phenotype
A-1	5	ll	hl	hl	hl	ll	ll	ll	75	55		
A-2	3	ll	hl	hl	hl	hl	hl	ll	94	71		
A-3	2	ll	ll	ll	ll	hl	hl	hl	66	0		
A-4	3	ll	ll	ll	ll	ll	ll	hl	92	0		
B-1	1	ll	hh	hh	hh	hh	hh	ll			7	R
B-2	4	ll	hh	hh	hh	ll	ll	ll			41	R
B-3	1	ll	hh	hh	ll	ll	ll	ll			23	R
B-4	2	ll	ll	ll	hh	hh	hh	hh			28	S
B-5	5	ll	ll	ll	ll	hh	hh	hh			31	S
B-6	2	ll	ll	ll	ll	ll	hh	hh			27	S
B-7	2	ll	ll	ll	ll	ll	ll	hh			30	S

*hh* homozygous for the *S*. *habrochaites* allele, *ll* homozygous for *S. lycopersicum* allele, *hl* heterozygous

*R* resistant, *S* susceptible

**Fig. 1:**
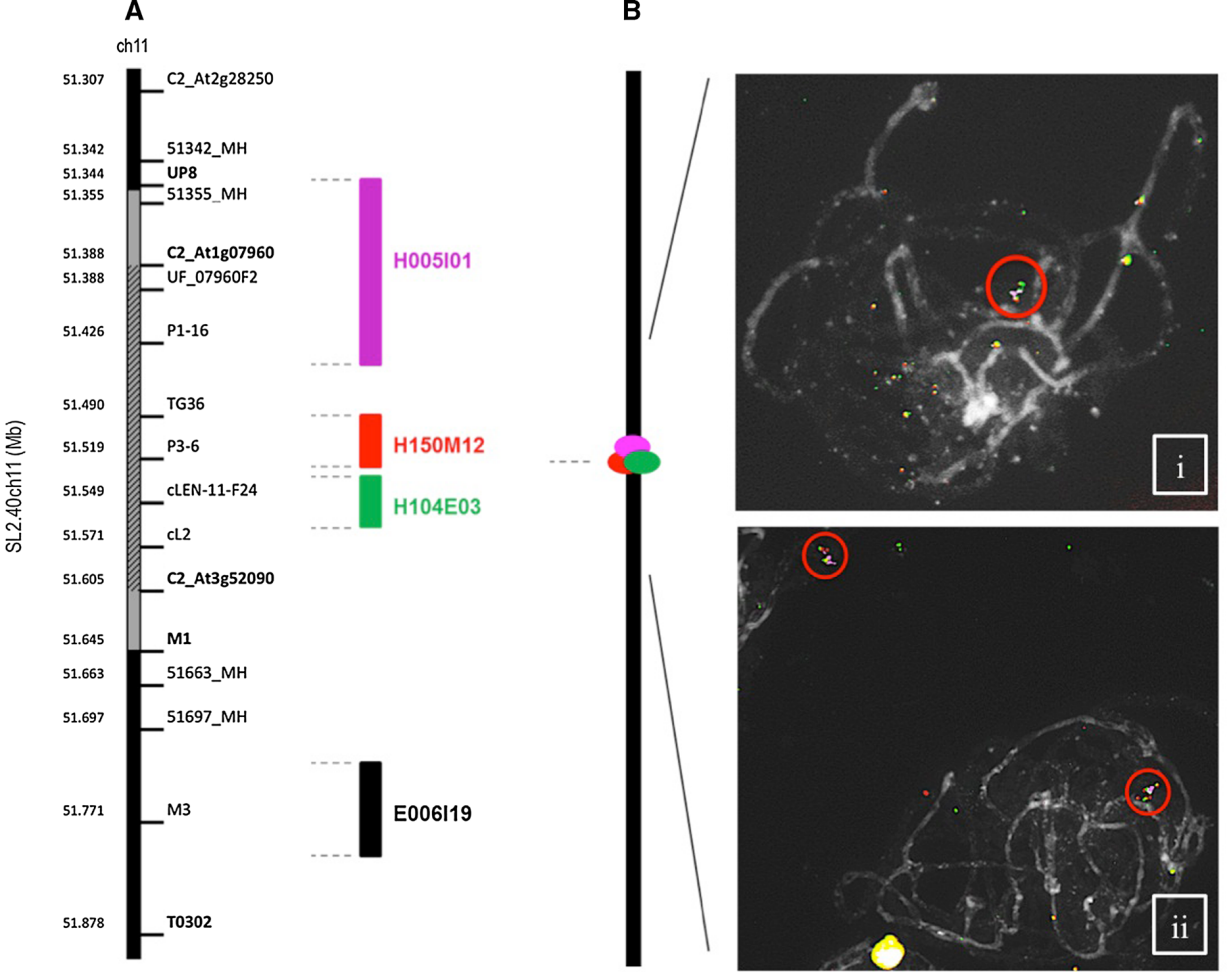
Genetic map of chromosome 11 (part). **A** Map position of the *Ty*-*2* gene is shown (*gray box* between markers UP8 and M1) and the region where suppression of recombination was identified (*shaded region* between markers C2_At1g07960 and C2_At3g52090). Bacterial artificial chromosome (BAC)s selected for fluorescence in situ hybridization (FISH) are shown in *color blocks*. **B** Schematic drawing of arrangements of BACs observed in FISH experiments. FISH images showing BAC signals in F_2_ plants *i* homozygous for the susceptible *S. lycopersicum* “Moneymaker” alleles and *ii* for the *S. habrochaites* alleles of loci in the *Ty*-*2* introgression. Overlapping BACs are observed for both genotypes. (Color figure online)

In Spring 2011, Phase II screening of approximately 7,000 additional plants identified 127 recombinants for the C2_At1g07960 and T0302 marker interval, but only 26 of these had recombinations above marker M1 (Table 2). Of those 26 recombinations, 24 cross-overs occurred between the markers C2_At3g52090 and M1, and the other two crossovers occurred between P1–16 and TG36. Selection for homozygosity of the recombined introgression of these 26 individuals resulted in three genotypic categories of RILs that were evaluated in Fall 2011. All Category I and II RILs containing the upper portion of the introgression were resistant, while all Category III RILs lacking this region were susceptible (Table 2), confirming the location of *Ty*-*2* above M1. Subsequent development and testing of additional markers between C2_At2g28250 and C2_At1g07960 determined that the upper end of the *S. habrochaites* introgression is likely between UP8 and C2_At1g07960 (Fig. 1). Thus, our results map *Ty*-*2* to the approximately 300,000-bp region between UP8 and M1.

**Table 2:** Genotype and phenotype of 26 recombinant inbred lines (RILs) identified from Phase II recombinant screening

Category	No. RILs	Molecular marker										Phenotype
		UP8	C2_At1g07 960	P1-16	TG36	cLEN-11-F24	cL1	cL2	C2_At3g52090	M1	T0302	
I	13	ll	hh	hh	hh	hh	hh	hh	hh	ll	ll	R
II	2	ll	hh	hh	hl	hl	hl	hl	hl	ll	ll	R
III	11	ll	ll	ll	ll	ll	ll	ll	ll	hh	hh	S

*hh* homozygous for *S*. *habrochaites* alleles, *ll* homozygous for *S. lycopersicum*alleles, *hl* heterozygous alleles

*R* resistant, *S* susceptible

### Skewed allele frequency in the *Ty*-*2* introgression

During the Phase II development of RILs, an interesting segregation pattern was obtained from progeny of the two Category II recombinants in the marker interval between P1–16 to TG36 (Table 2). Each of these recombinants was homozygous for *S. habrochaites* alleles (*hh*) between markers C2_At1g07960 and P1-16 and heterozygous (*hl*) at markers TG36 to M1. To generate RILs, progeny of these two individuals were screened with marker M1 to select plants homozygous for the *S. lycopersicum* allele (*ll*). For each recombinant, 48 seedlings were screened and several individuals were selected. Segregation at M1 had an acceptable fit to a 1:2:1 ratio (1*hh*:2*hl*:1*ll*). However, subsequent screening of these individuals with additional markers between P1–16 and M1 showed that all selected plants, although homozygous for the *S. lycopersicum* allele at M1, remained heterozygous for all markers tested in the TG36 to C2_At3g52090 interval (Table 2). Further screening of nearly 100 progeny from each of the two Category II recombinants confirmed this result, and no progeny were identified that were homozygous for the *S. lycopersicum* alleles in the TG36 to C2_At3g52090 interval. Within this interval, the allele frequency of *hh*:*hl* segregated in a 1:3 ratio. Although all progeny of these two recombinants showed clear TYLCV resistance, the failure to recover homozygous *S. lycopersicum* alleles between the TG36 to C2_At3g52090 interval from the genotyping of 200 plants prevented the further narrowing of the *Ty*-*2* region.

### Suppression of recombination in the *Ty*-*2* introgression

In summary, Phase I and Phase II screening of approximately 11,000 progeny identified 157 recombinants for the approximately 500,000-bp region between C2_At1g07960 and T0302. Only 29 of these crossovers occurred above marker M1; of these, 27 occurred in the approximately 35,000-bp region between C2_At3g52090 and M1, and only two were in the approximately 60,000-bp region between P1–16 and TG36. No recombinants were found between TG36 and C2_At3g52090, indicating the suppression of recombination.

To clarify whether the suppression was population-dependent, a recombinant screening was carried out in Wageningen (the Netherlands) in another F_2_ population derived from a round tomato F_1_ hybrid. In this F_2_ population, markers from 51355_MH through T0302 segregated (Fig. 1), showing that a large introgression of *S. habrochaites* is present in the commercial hybrid carrying the *Ty*-*2* gene. The presence of the *Ty*-*2*-conferred resistance was confirmed by challenging 110 F_2_ plants with TYLCV and genotyping them with markers between 51355_MH and T0302. Among the 110 plants tested, 25 showed TYLCV symptoms similar to the susceptible control, MM, and were homozygous for the susceptible allele at all tested markers. The other 85 plants showing slight or no symptoms were scored as resistant. Resistant plants were either homozygous or heterozygous for *S. habrochaites* alleles at all tested markers. Thus, there was no skewing of allele frequency in the region between 51355_MH and T0302 in this F_2_ population. By screening an additional 1900 plants of this F_2_ population with markers UF_07960F2 and T0302, 18 recombinants were identified (data not shown) and all recombination events occurred downstream of the marker C2_At3g52090 (Fig. 1), confirming a severe suppression of recombination in the region between markers C2_At1g07960 and C2_At3g52090.

As with the *Ty*-*1* introgression (Verlaan et al. 2011), we hypothesized that differences in chromosome structure between the two parental lines might be the cause for the suppression of recombination. We previously showed that fluorescent in situ hybridization (FISH) can be used as a molecular tool to reveal inversions or chromosomal rearrangements among several *Solanum* species (Szinay et. al. 2012). Therefore, we applied FISH analysis in order to visualize the chromosome structure of the *Ty*-*2* introgression. Four BACs located within the 300 kb *S. habrochaites* introgression were selected (Fig. 1) and labeled for FISH as described in Verlaan et al. (2011). Unfortunately, FISH images showed overlapping fluorescing foci from their corresponding BACs, indicating that this 300-kb region is too small for resolution using the FISH technique.

### Differential expression of the candidate genes

**Fig. 2:**
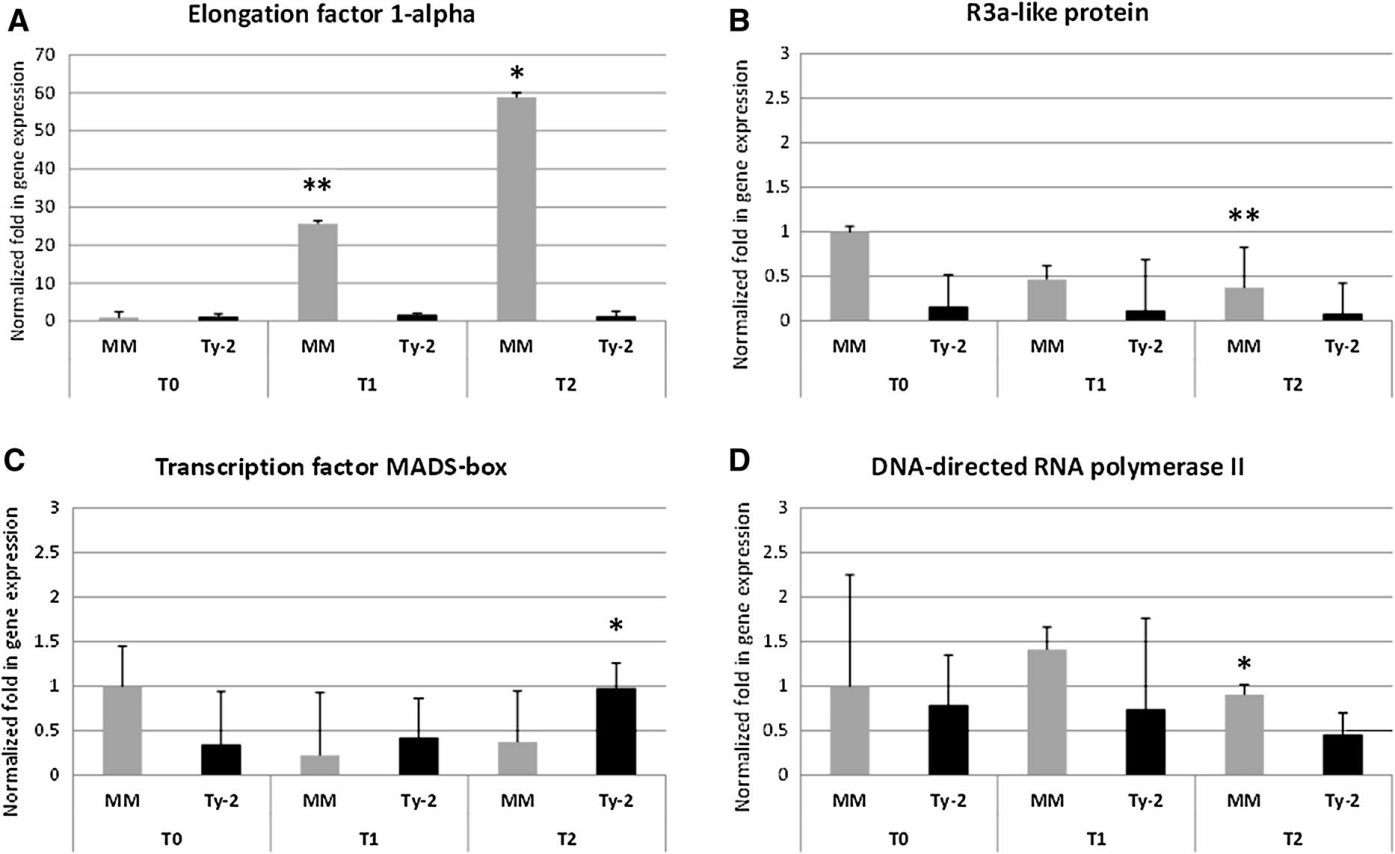
Relative expression of candidate genes. Normalized fold in gene expression of differentially expressed candidate genes as determined by RT-PCR; **A** Elongation factor 1-alpha, **B** R3a-like protein, **C** Transcription factor MADS-box, **D** DNA-directed RNA polymerase. Time points are shown in the *x*-axis; T0, T1, and T2 (0, 9, and 20 days after TYLCV inoculation). Values are normalized against the Moneymaker T0 sample; *bars* represent means and standard deviation of three biological replicas. *Asterisks* above the *bars* represent significant differences between genotypes per time point (*P < 0.05, **P < 0.01)

Within the 300-kb *Ty*-*2* region, 35 genes were annotated in the tomato sequence version SL2.40, Sol Genomics Network (Table S2). In order to examine the effects of TYLCV infection on the expression of the predicted genes in the *Ty*-*2* region, relative expression levels were quantified at 0, 9, and 20 days after TYLCV inoculation. Resistant plants carrying the *Ty*-*2* introgression and susceptible MM plants were sampled. Priority was given to genes expressed according to the RNAseq coverage information (Sol Genomics Network), and transcript levels of 25 out of the 35 predicted genes were quantified by RT-PCR (Table S3). Using ubiquitin as the housekeeping gene, three predicted genes were shown to be differentially expressed in the *Ty*-*2* plants compared to the susceptible genotype and upon TYLCV inoculation (Fig. 2). These are Solyc11g069700.1, encoding an elongation factor 1-alpha; Solyc11g069770.1, encoding a transcription factor of MADS-box family; and Solyc11g069930.1, encoding an *R3a*-like resistance protein. Among these genes, the elongation factor 1-alpha and the disease resistance protein *R3a*-like showed lower expression in the resistant genotype. Twenty days after virus infection, relative amount of transcripts of the elongation factor in the susceptible genotype was over 40 times higher than in the resistant plants (Fig. 2A), and across all time points, the expression level of this gene in the resistant plants remained very low. Expression of the predicted *R3a*-like homolog in the resistant plants remained almost four times lower than in the susceptible genotype (Fig. 2B) across all time points. In contrast, expression of the transcription factor MADS-box in the resistant plants was 2.5 times higher than in the susceptible phenotype 20 days after TYLCV infection (Fig. 2C). In addition to these three genes, Solyc11g069910.1, the gene encoding a DNA-directed RNA polymerase II, showed a down-regulation in the *Ty*-*2* line upon TYLCV inoculation, almost 2 times lower than in the susceptible MM plants (Fig. 2D).

### Silencing of the differentially expressed candidate genes

**Fig. 3:**
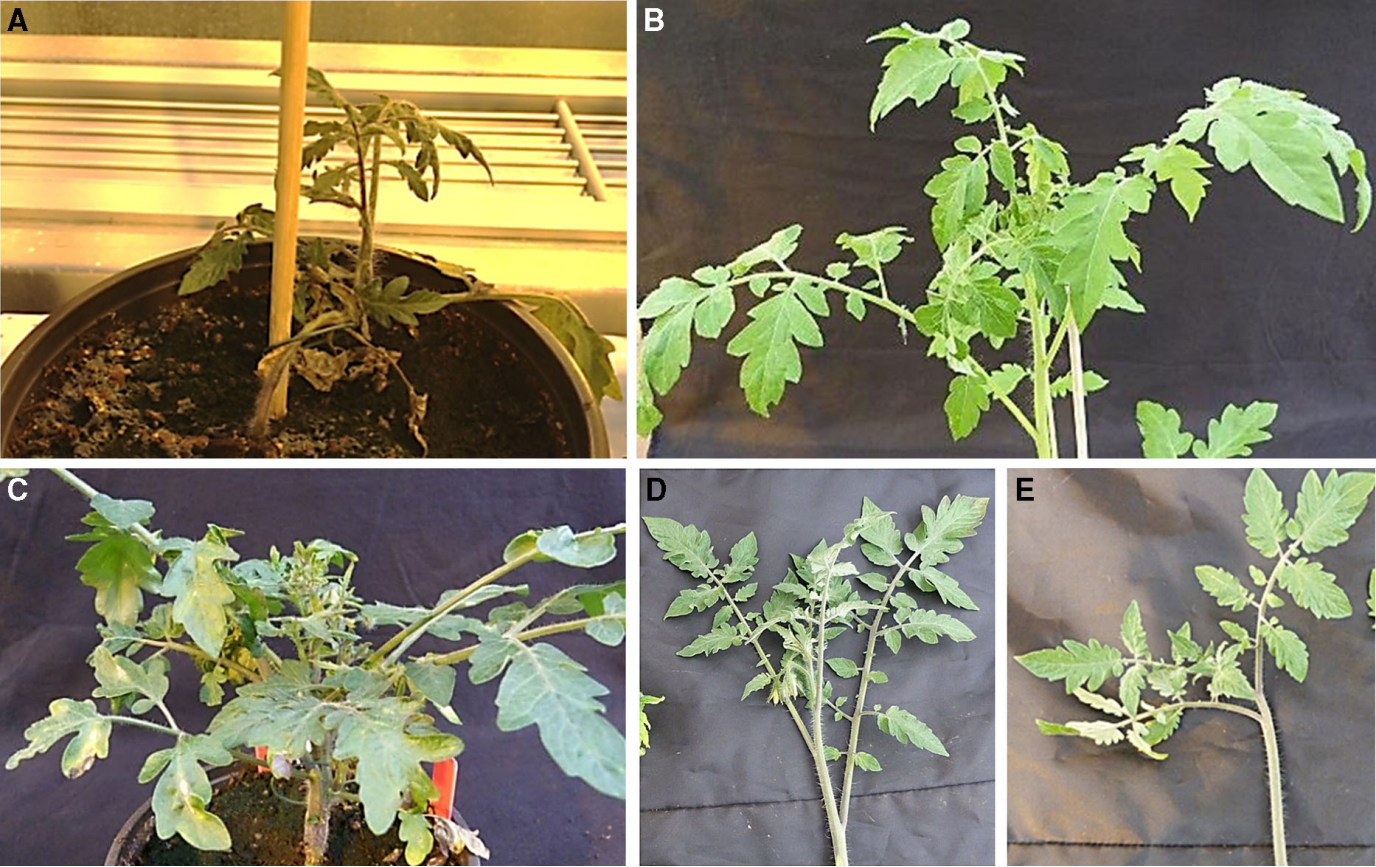
Candidate genes silencing effects. Pictures were taken from resistant plants carrying *Ty*-*2*. Targeting three candidate genes showed abnormal phenotypes: **A** Elongation factor 1-alpha. Targeting this gene had a lethal effect. **B** Transcription factor MADS-box. *Yellowish* leaves and smaller and weaker plants were observed upon TYLCV infection. **C** DNA-directed RNA polymerase II. A stunted plant with shorter internodes and curled small leaves was observed. **D** *R3a* homologs. Resistance was not compromised; no phenotype was observed. **E** TRV-empty vector control plant. (Color figure online)

To determine the implication of the candidate genes on TYLCV resistance, specific VIGS constructs (Table S4) were designed to silence these four candidate genes in MM and the line carrying the *Ty*-*2* gene. Two weeks after infiltration with TRV vector for gene silencing, plants were challenged with TYLCV. Plants infiltrated with TRV vectors but noninfected with TYLCV and plants infiltrated with an empty (EV) TRV vector were used as controls. Except for *R3a*-like genes, plants infiltrated with TRV vectors targeting these genes all showed an abnormal phenotype when compared to the control plants (Fig. 3). Silencing the elongation factor 1-alpha had a lethal effect (Fig. 3A); silencing the transcription factor MADS-box resulted in plants with yellowish leaves (Fig. 3B); and silencing the DNA-directed RNA polymerase II protein led to stunted plants with smaller and curled leaves (Fig. 3C). These phenotypes were observed in plants of the *Ty*-*2* line and MM before TYLCV inoculation, thus determined by the silencing of the target gene itself rather than induced by TYLCV infection.

Candidate genes in the *Ty*-*2* region mapped in this study include five genes encoding CC-NBS-LRR proteins, of which two are *R3a*-like. *R3a* is a member of the *R3* complex locus on chromosome 11 of potato, which confers race-specific resistance to the oomycete *Phytophtora infestans* (Huang et al. 2005). To determine whether these *R3a* homologs are required for *Ty*-*2*-mediated resistance to TYLCV, a VIGS construct (VG930) targeting both Solyc11g069670.1 and Solyc11g069930.1 was generated (Figure S1). After silencing, plants were TYLCV inoculated and subsequently monitored for the development of symptoms. Until 45 days after virus infection, no viral symptoms were recorded (Fig. 3D). The phenotype observed was similar to that displayed by the control plants (inoculated with an empty TRV vector and infiltrated only with TRV silencing vector). Our data indicate that silencing of the *R3a* homologs does not compromise the resistance conferred by *Ty*-*2*.

## Discussion

Recombination suppression is a common phenomenon in genomic regions introgressed from wild tomato species (Ji et al. 2007b, 2009a). Therefore, it was not surprising to observe this in the introgressed segment containing the *Ty*-*2* gene. In a previous study, the *Ty*-*2* gene was delimited to a region between the markers C2_At2g28250 and T0302, a distance of at least 500 kb (Ji et al. 2009a). Although approximately 11,000 plants were genotyped in the present study, only 157 recombinants within this region were obtained. These recombinants did allow the further delimiting of *Ty*-*2* to a shorter region spanned by markers UP8 and M1 of ≈300,000 bp. The reason for this suppression is unknown, but perhaps there is an inversion as there was on chromosome 6 in a region where *Ty*-*1* and *Ty*-*3* have been mapped (Verlaan et al. 2011). Unfortunately, FISH was not powerful in this case to visualize any potential chromosomal rearrangements. Alternatively, a region of duplication or a cold spot for recombination could also explain the suppression of recombination. Previous studies have shown that recombination frequency is positively related to the length of alien segments and that, in some cases, cross direction also has significant impact on the frequency of recombination (Canady et al. 2006; Li et al. 2010). In order to increase the frequency of recombination in the *Ty*-*2* region, it would be helpful to use populations derived from lines with a larger *Ty*-*2* introgressed segment. In case that chromosomal rearrangement is present in *S. habrochaites*, the best option would be to use an intraspecific cross with susceptible *S. habrochaites* accession to facilitate the cloning of *Ty*-*2*.

Whatever the reason for the suppression of recombination, the inability to further reduce the size of the introgressed chromosome segment has an important impact on practical breeding for two reasons. First, there is the possibility of linkage drag. No reports of linkage drag associated with *Ty*-*2* have been published to date, but in large fruited tomato germplasm, a rough blossom scar where teratomas emerge has been associated with resistance from *Ty*-*2* (Ryohei Arimoto, personal communication). Our lines with the shortest introgressions need to be tested to determine whether this problem has been eliminated. Secondly, the large chromosome segment introgressed from wild species can hamper combining important genes in *cis*. The fusarium wilt race 2 resistance gene, *I*-*2*, has been cloned (Simons et al. 1998) and is located on chromosome 11 at approximately 52 Mb according to version SL 2.40 of the tomato genome assembly. Considering that this locus is more than 400,000 bp below *Ty*-*2*, it should not be a significant problem to combine the two genes in *cis*, although a directed effort will be needed.

Due to the suppression of recombination and skewing of allele frequencies, it is difficult to further delimit *Ty*-*2* into a smaller region in order to pinpoint the candidate. Therefore, we have performed gene expression and VIGS experiments in order to predict potential candidates for *Ty*-*2*. There are 35 genes predicted in the target region; among these are genes involved in plant defense mechanisms or signaling pathways against viruses or other pathogens, such as ABC transporters, kinases, receptor-like proteins or cytochrome P450 (Krattinger et al. 2009; Tena et al. 2011; Larkan et al. 2013; Howe et al. 2000). In order to more accurately determine the potential candidates for *Ty*-*2,* we have performed gene expression and VIGS experiments. Of the 35 genes predicted in the target region, 25 were checked for expression and 4 out of these showed to be differentially expressed in the *Ty*-*2* line upon TYLCV infection. These genes encode for an elongation factor 1-alfa, a *R3a*-like protein, a DNA-directed RNA polymerase II, and a transcription factor of the MADS-box family.

Host translation elongation factors are involved in the multiplication of viruses in multiple organisms (Lai 1998). Elongation factor 1-alpha has been found to interact with several viral proteins (Buck 1999; Thivierge et al. 2008) and recently recorded in a metabolite profile of a TYLCV resistant line upon TYLCV infection (Moshe et al. 2012). Matsuda and Dreher (2004) suggested EF1-alpha to enhance the translation of *Turnip yellow mosaic virus* RNA; therefore, decreased amounts of gene products might prevent or interfere with viral replication, thus leading to resistance. We observed a reduced expression of the EF-1 alpha on *Ty*-*2*-resistant plants, before and after TYLCV infection. However, silencing this gene led to the collapse of the plants, preventing us to elucidate its implication on the *Ty*-*2*-mediated resistance.

The most interesting altered phenotype was shown by silencing Solyc11g069910.1, the gene encoding a DNA-directed RNA polymerase II (Pol II). DNA-dependent RNA polymerases mediate epigenetic silencing as a resistance mechanism against geminiviruses. DNA-dependent RNA polymerases IV and V (and indirectly Pol II) are involved in the RNA-directed DNA methylation (RdDM) process, which can lead to transcriptional silencing, not only of viral invading DNA but also of host nuclear genes, transposons, and repetitive elements (Carr et al. 2010; Haag and Pikaard 2011). It might be possible that this gene is targeted by the virus, interfering with the RdDM process and causing epigenetic changes in the host and/or viral DNA, consequently producing TYLCV-like symptoms, e.g., small and curling leaves of stunting plants.

The MADS-box family is described to mainly play fundamental roles in plant development (Kaufmann et al. 2009), but it is also involved in various stress-related processes (Lee et al. 2008). Silencing Solyc11g069770.1, a transcription factor MADS-box, led to yellowish leaves. Although it is speculative, our results may suggest that TYLCV suppresses the expression of the transcription factor MADS-box leading to yellowish leaves.

In the *Ty*-*2* region, three genes are predicted to encode CC-NBS-LRR, NBS-LRR, and NBS resistance proteins. Additionally, two genes coding for a disease resistance *R3a*-like protein (fragment) and disease resistance R3a-like protein are predicted and each contains the NB-ARC dominion. *Ty*-*2* is a dominant resistance gene, and to date, most of the cloned dominant resistance genes encode proteins containing the conserved NB-ARC domain, making these genes likely candidates. However, silencing both *R3a*-like homologs did not compromise the resistance conferred by *Ty*-*2*, suggesting that this gene may not belong to a NBS gene family.

*Ty*-*2* has shown complete dominance for TYLCV resistance (Ji et al. 2009a), but has been ineffective against some TYLCV strains and against bipartite begomoviruses (Mejía et al. 2005). The *Ty*-*3* locus has generally shown less dominance, but a wider range of resistance against TYLCV strains and bipartite begomoviruses (Ji et al. 2007a). Hybrids with the heterozygous combination of both genes may prove to be effective and durable against a wide array of begomoviruses. Although *Ty*-*2* alone provided no resistance to bipartite begomoviruses in Guatemala, pyramiding *Ty*-*2* and *Ty*-*3* together provided a higher level of resistance than *Ty*-*3* alone (Mejía et al. 2010). Vidavski (2007) and Vidavski et al. (2008) also showed that combining different begomovirus resistance genes can have unanticipated synergistic effects, and the combination of *Ty*-2 with other genes should be tested further in this regard. Tightly linked PCR markers can be used to effectively tag these TYLCV resistance genes, and expedite the process of pyramiding these resistance genes of various origins into a single elite genotype, thus improving the resistance to TYLCV as well as broadening the resistance against a wider range of begomoviruses.

## Electronic supplementary material

Below is the link to the electronic supplementary material.

Supplementary material 1 (DOCX 18 kb)

Supplementary material 2 (DOCX 17 kb)

Supplementary material 3 (DOCX 16 kb)

Supplementary material 4 (DOCX 16 kb)

Supplementary material 5 Figure S1. Target regions for silencing the *R3a* homologs in tomato chromosome 11. Nucleotide sequence alignments of predicted R3a homologs in the *Ty-2* region are shown: Disease resistance protein R3a-like fragment (Solyc11g069670.1), Disease resistance protein *R3a*-like protein (Solyc11g069930.1), and TRV-based VIGS construct VG930. Regions highlighted in black represent sequences targeted for VIGS for each predicted gene. cDNA sequences were obtained from SGN public database (TIFF 68 kb)

## References

[CR1] Anbinder I, Reuveni M, Azari R, Paran I, Nahon S, Shlomo H, Chen L, Lapidot M, Levin I (2009). Molecular dissection of tomato leaf curl virus resistance in tomato line TY172 derived from *Solanum peruvianum*. Theor Appl Genet.

[CR2] Antignus Y, Nestel D, Cohen S, Lapidot M (2001). Ultraviolet-deficient greenhouse environment affects whitefly attraction and flight-behavior. Environ Entomol.

[CR3] Buck KW (1999). Replication of tobacco mosaic virus RNA. Philos Trans R Soc Lond B Biol Sci.

[CR4] Canady MA, Ji Y, Chetelat RT (2006). Homeologous recombination in Solanum lycopersicoides introgression lines of cultivated tomato. Genetics.

[CR5] Carr JP, Lewsey MG, Palukaitis P (2010). Signaling in induced resistance. Adv Virus Res.

[CR6] Cohen S, Lapidot M, Czosnek H (2007). Appearance and expansion of TYLCV: a historical point of view. Tomato yellow leafcurl virus disease.

[CR7] Griffiths PD, Scott JW (2001). Inheritance and linkage of tomato mottle virus resistance genes derived from *Lycopersicon chilense* accession LA 1932. J Am Soc Hort Sci.

[CR8] Haag JR, Pikaard CS (2011). Multisubunit RNA polymerases IV and V: purveyors of non-coding RNA for plant gene silencing. Nat Rev Mol Cell Biol.

[CR9] Hanson PM, Bernacchi D, Green S, Tanksley SD, Muniyappa V, Padmaja AS, Chen H, Kuo G, Fang D, Chen J (2000). Mapping a wild tomato introgression associated with tomato yellow leaf curl virus resistance in a cultivated tomato line. J Am Soc Hort Sci.

[CR10] Hanson PM, Green SK, Kuo G (2006). *Ty*-*2*, a gene on chromosome 11 conditioning geminivirus resistance in tomato. Rep Tomato Genet Coop.

[CR11] Horowitz AR, Denholm I, Morin S, Czosnek H (2007). Resistance of the TYLCV whitefly vector *Bemisia tabaci* to insecticides. Tomato yellow leafcurl virus disease.

[CR12] Howe GA, Lee GI, Itoh A, Li L, DeRocher AE (2000). Cytochrome P450-dependent metabolism of oxylipins in tomato. Cloning and expression of allene oxide synthase and fatty acid hydroperoxide lyase. Plant Physiol.

[CR13] Huang S, Van Der Vossen EA, Kuang H, Vleeshouwers VG, Zhang N, Borm TJ, Van Eck HJ, Baker B, Jacobsen E, Visser RG (2005). Comparative genomics enabled the isolation of the R3a late blight resistance gene in potato. Plant J.

[CR14] Hutton SF, Scott JW, Schuster DJ (2012). Recessive resistance to *Tomato yellow leaf curl virus* from the tomato cultivar Tyking is located in the same region as *Ty*-5 on chromosome 4. HortScience.

[CR15] Ji Y, Schuster DJ, Scott JW (2007). *Ty*-*3*, a begomovirus resistance locus near the *Tomato yellow leaf curl virus* resistance locus *Ty*-*1* on chromosome 6 of tomato. Mol Breed.

[CR16] Ji Y, Scott JW, Hanson P, Graham E, Maxwell DP, Czosnek H (2007). Sources of resistance, inheritance, and location of genetic loci conferring resistance to members of the tomato-infecting begomoviruses. Tomato yellow leaf curl virus disease: management, molecular biology, breeding for resistance.

[CR17] Ji Y, Salus MS, van Betteray B, Smeets J, Jensen KS, Martin CT, Mejía L, Scott JW, Havey MJ, Maxwell DP (2007). Co-dominant SCAR markers for detection of the *Ty*-*3* and *Ty*-*3a* loci from *Solanum chilense* at 25 cM of chromosome 6 of tomato. Rep Tomato Genet Coop.

[CR18] Ji Y, Scott JW, Maxwell DP, Schuster DJ (2008). *Ty*-*4*, a tomato yellow leaf curl virus resistance gene on chromosome 3 of tomato. Rep Tomato Genet Coop.

[CR19] Ji Y, Scott JW, Schuster DJ (2009). Toward fine mapping of the *Tomato yellow leaf curl virus* resistance gene *Ty*-*2* on chromosome 11 of tomato. HortScience.

[CR20] Ji Y, Scott JW, Schuster DJ, Maxwell DP (2009). Molecular mapping of *Ty*-*4*, a new *Tomato Yellow Leaf Curl Virus* resistance locus on chromosome 3 of tomato. J Am Soc Hort Sci.

[CR21] Kalloo G, Banerjee MK (1990). Transfer of tomato leaf curl virus resistance from *Lycopersicon hirsutum* f. *glabratum* to *L. esculentum*. Plant Breed.

[CR22] Kaufmann K, Muino JM, Jauregui R, Airoldi CA, Smaczniak C, Krajewski P, Angenent GC (2009). Target genes of the MADS transcription factor SEPALLATA3: integration of developmental and hormonal pathways in the Arabidopsis flower. PLoS Biol.

[CR23] Krattinger SG, Lagudah ES, Spielmeyer W, Singh RP, Huerta-Espino J, McFadden H, Bossolini E, Selter LL, Keller B (2009). A putative ABC transporter confers durable resistance to multiple fungal pathogens in wheat. Science.

[CR24] Lai M (1998). Cellular factors in the transcription and replication of viral RNA genomes: a parallel to DNA-dependent RNA transcription. Virology.

[CR25] Larkan N, Lydiate D, Parkin I, Nelson M, Epp D, Cowling W, Rimmer S, Borhan M (2013). The Brassica napus blackleg resistance gene LepR3 encodes a receptor-like protein triggered by the Leptosphaeria maculans effector AVRLM1. New Phytol.

[CR26] Lee S, Woo Y-M, Ryu S-I, Shin Y-D, Kim WT, Park KY, Lee I-J, An G (2008). Further characterization of a rice AGL12 group MADS-box gene, OsMADS26. Plant Physiol.

[CR27] Levin I, Karniel U, Fogel D, Reuveni M, Gelbart D, Evenor D, Chen L, Nahon S, Shlomo H, Machbosh Z, Lapidot M (2013) Cloning and analysis of the *Tomato yellow leaf curl virus* resistance gene *Ty*-*5*. In: Proceedings of the tomato breeders roundtable, Chaing-Mai, Thailand. http://tgc.ifas.ufl.edu/2013/abstracts/LevinAbstract%20TBRT%202013.pdf

[CR28] Li W, Royer S, Chelelat RT (2010). Fine Mapping of *ui6.1*, a gametophytic factor controlling pollen-side unilateral incompatibility in interspecific *Solanum* Hybrids. Genetics.

[CR29] Liu Y, Schiff M, Dinesh-Kumar S (2002). Virus-induced gene silencing in tomato. Plant J.

[CR30] Matsuda D, Dreher TW (2004). The tRNA-like structure of Turnip yellow mosaic virus RNA is a 3′-translational enhancer. Virology.

[CR31] Mejía L, Teni RE, Vidavski F, Czosnek H, Lapidot M, Nakhla MK, Maxwell DP (2005). Evaluation of tomato germplasm and selection of breeding lines for resistance to begomoviruses in Guatemala. Acta Hort.

[CR32] Mejía L, Teni RE, García BE, Fulladolsa AC, Méndez L, Melgar S, Maxwell DP (2010). Preliminary observations on the effectiveness of five introgressions for resistance to begomoviruses in tomatoes. TGC Report.

[CR33] Moshe A, Pfannstiel J, Brotman Y, Kolot M, Sobol I, Czosnek H, Gorovits R (2012) Stress responses to tomato yellow (leaf curl virus TYLCV) infection of resistant and susceptible tomato plants are different. Metabolomics 2153-0769

[CR34] Pico B, Diez MJ, Nuez F (1996). Viral diseases causing the greatest economic losses to the tomato crop. 2. The tomato yellow leaf curl virus—a review. Sci Hort.

[CR35] Schmitz G, Tillmann E, Carriero F, Fiore C, Cellini F, Theres K (2002). The tomato blind gene encodes a MYB transcription factor that controls the formation of lateral meristems. Proc Natl Acad Sci USA.

[CR36] Scott JW, Razdan MK, Mattoo AK (2007). Breeding for resistance to viral pathogens. Genetic improvement of solanaceous crops.

[CR37] Scott JW, Agrama HA, Jones JP (2004). RFLP-based analysis of recombination among resistance genes to fusarium wilt races 1, 2 and 3 in tomato. J Am Soc Hort Sci.

[CR38] Simons G, Groenendijk J, Wijbrandi J, Reijans M, Groenen J, Diergaarde P, Van der Lee T, Bleeker M, Onstenk J, de Both M, Haring Jurriaan Mes M, Cornelissen B, Zabeau M, Vos P (1998). Dissection of the fusarium *I2* gene cluster in tomato reveals six homologs and one active gene copy. Plant Cell.

[CR39] Szinay D, Wijnker E, van den Berg R, Visser RG, de Jong H, Bai Y (2012). Chromosome evolution in Solanum traced by cross-species BAC-FISH. New Phytol.

[CR40] Tena G, Boudsocq M, Sheen J (2011). Protein kinase signaling networks in plant innate immunity. Curr Opin Plant Biol.

[CR41] Thivierge K, Cotton S, Dufresne PJ, Mathieu I, Beauchemin C, Ide C, Fortin MG, Laliberté J-F (2008). Eukaryotic elongation factor 1A interacts with *Turnip mosaic virus* RNA-dependent RNA polymerase and VPg-Pro in virus-induced vesicles. Virology.

[CR42] Thornton B, Basu C (2011). Real-time PCR (qPCR) primer design using free online software. Biochem Mol Biol Educ.

[CR43] Verlaan MG, Szinay D, Hutton SF, de Jong H, Kormelink R, Visser RGF, Scott JW, Bai YL (2011). Chromosomal rearrangements between tomato and *Solanum chilense* hamper mapping and breeding of the TYLCV resistance gene *Ty*-*1*. Plant J.

[CR44] Verlaan MG, Hutton SF, Ibrahem RM, Kormelink R, Visser RG, Scott JW, Edwards JD, Bai Y (2013). The tomato yellow leaf curl virus resistance genes Ty-1 and Ty-3 are allelic and code for DFDGD-class RNA–dependent RNA polymerases. PLoS Genet.

[CR45] Vidavski FS, Czosnek H (2007). Exploitation of resistance genes found in wild tomato species to produce resistant cultivars; pile up of resistant genes. Tomato yellow leaf curl virus disease: management, molecular biology, breeding for resistance.

[CR46] Vidavski F, Czosnek H, Gazit S, Levy D, Lapidot M (2008). Pyramiding of genes conferring resistance to *Tomato yellow leaf curl virus* from different wild tomato species. Plant Breed.

[CR47] Zamir D, Eksteinmichelson I, Zakay Y, Navot N, Zeidan M, Sarfatti M, Eshed Y, Harel E, Pleban T, Vanoss H, Kedar N, Rabinowitch HD, Czosnek H (1994). Mapping and introgression of a tomato yellow leaf curl virus tolerance gene, *Ty*-*1*. Theor Appl Genet.

