# Supplementary material for: Fine mapping of the tomato yellow leaf curl virus resistance gene Ty-2 on chromosome 11 of tomato
Source: Mol Breed. 2014 Mar 28;34(2):749–60. doi: 10.1007/s11032-014-0072-9 (PMC4092234; doi:10.1007/s11032-014-0072-9)
Supplement: Supplementary file 1 — Supplementary material 1 (DOCX 18 kb) [file 11032_2014_72_MOESM1_ESM.docx]

**Table S1.** Molecular markers used for mapping *Ty*-2 on tomato chromosome 11

| Marker | Physical  position (Mb)^a^ | Primer^b^ | Restriction enzyme | Source of  primers^c^ |
| --- | --- | --- | --- | --- |
| C2_At2g28250 | SL2.40ch11: 51.307 | F-AGACTTCATCATCGTCATGTGGTTCCG  R-TTTGGAGGTGCTTTGCCATACCAAG | *DdeI* | SGN |
| 51342_MH | SL2.40ch11: 51.342 | F-ACCCCCACTCCATGATATT  R-GCTGGAGAAGCTGGACCATA | None | This study |
| UP8 | SL2.40ch11: 51.344 | F-GCGCTGCTAGACATTTCGAT  R-CTGAAGTTGCTTGAATGCTCA | None | This study |
| 51355_MH | SL2.40ch11: 51.355 | F-GCTAGAGCTTTCAAATCACTCTCAA  R-GCTCATTGGCATTCACCTTCT | *FspBI* | This study |
| 51372_MH | SL2.40ch11: 51.372 | F-GTTGGGAGCAACTCAGGTGA  R-CCAGCACTAGGACAGCTTCC | *Eco32I* | This study |
| UP15 | SL2.40ch11: 51.381 | F-TCTCAAAGCGTTGATCGTTG  R- GCTTGCTCTTGTTGGTCTCC | *EcoRV* | This study |
| UF_07960F2 | SL2.40ch11: 51.388 | F-CGTGCCACCCCTTCATAATA  R-CCCTTGCGAGGAAAATACAG | *BanI* | This study |
| C2_At1g07960 | SL2.40ch11: 51.387 | F-AAAGCCATTGTTACCGTCTCCGTG  R-AGCCATAAGTGGTGTGGAGGACTT | *RsaI* | Ji et al. (2009) |
| P1-16 | SL2.40ch11: 51.426 | F-CACACATATCCTCTATCCTATTAGCTG  R-CGGAGCTGAATTGTATAAACACG | None | This study |
| P1-19 | SL2.40ch11: 51.432 | F-TAACACCAAATCGCGTCTGA  R-TTGGGAAAACTATAGCATCG | *AseI* | This study |
| TG36 | SL2.40ch11: 51.490 | F-AACCACCACAAGAAAGATCCC  R-TCCTGAAATGGAAGATTGCC | *RsaI* | Schmitz et al. (2002) |
| T0386_MH | SL2.40ch11: 51.499 | F-CATTTGCTTTACTGCTAGTGTGC  R-GGTTGACCATCTCGAACTCC | None | This study |
| T0386A | SL2.40ch11: 51.503 | F-ATGCTGATGAAAGATTGGGCGCTG  R-TTAGGCTTTGGCTTCTCGACCACT | *HinfI* | Ji et al. (2009a) |
| P3-6 | SL2.40ch11: 51.519 | F-TGGTGTTTTGTGCGGTAAGA  R-TGAAATCGCATGTCCAAAGA | *HaeIII* | This study |
| P8687 | SL2.40ch11: 51.534 | F-TACCGTTGCGTAATCTAA  R-TTCCACTCAGCATCCCTA | *HinfI* | This study |
| P4-2 | SL2.40ch11: 51.543 | F-TCATTCACGGGGAAATTAGG  R-CAAGGGACCCAACTTTTTGA | *HinfI* | This study |
| cLEN-11-F24 | SL2.40ch11: 51.549 | F-TTATGGACAGCATGGTCCTCGGAA  R-GAAGTCTGGGAGCGATAGTAGTCT | *MnlI* | Ji et al. (2009) |
| cL1 | SL2.40ch11: 51.559 | F-ATTGCCTACATCTGGTTC  R-AAGATACCCACAAGACAA | *HaeIII* | This study |
| cL2 | SL2.40ch11: 51.571 | F-GGTAGGGATAAGGTCTGT  R-CCTTAGCCGTTACACTCT | *BclI* | This study |
| P7-8 | SL2.40ch11: 51.600 | F-TTGACCACGTTTTGGAAATG  R-GCAAGAAGACGCTTTTCGAT | *HindIII* | This study |
| C2_At3g52090 | SL2.40ch11: 51.605 | F-AGGGATACGAAGATCATGAATGCAGC  R-ACTCTTCAGATGATCAAGTTCCTTGTC | *None* | SGN |
| P8-8 | SL2.40ch11: 51.628 | F-AGTGGAACTTAATGGCTTTCC  R-CGCAATTGACGCATACATTC | *TaqI* | This study |
| 51632_MH | SL2.40ch11: 51.632 | F-GGCACTGATGGAGGAGAGTT  R-AGCTCACCTGTTGACCTTCA | *DraI* | This study |
| P8-11 | SL2.40ch11: 51.635 | F-CGACAGTGTTTTCACCAGCTC  R-ACCGAGTATGCACCACCAAT | *RsaI* | This study |
| M1 | SL2.40ch11: 51.645 | F-CGCTCGGGCAAATAGTTCGTAATGG  R-TTCATGGTCTAGAAATGTCCCCTGT | *BstUI* | This study |
| M2 | SL2.40ch11: 51.661 | F-TCAGGGAAGTCTATGTAAACGC  R-ATGTGGTAGATAGAAGGGAAGC | *HindIII* | This study |
| 51663_MH | SL2.40ch11: 51.663 | F-CCCTCTTGCTTAGTGGGTGA  R-ACGCTCCAAATCAGAGGTTG | *Hin6I* | This study |
| C2_At4g32930 | SL2.40ch11: 51.688 | F-TCCTCTTCCTATTGGCAAGGGC  R-TGGACACTCCCCCTTTTCATCATAC | *Cfr13I* | SGN |
| 51697_MH | SL2.40ch11: 51.697 | F-CCCTCAAACCCAAGTGCTTAC  R-CTCCAACTTTGCGACTGTTCT | *RsaI* | This study |
| 51752_MH | SL2.40ch11: 51.752 | F-ACTCTTGCTTCACTCCTTGGA  R-ACCATACCTCAACTTGGAAACA | *SspI* | This study |
| M3 | SL2.40ch11: 51.771 | F-TGAATGGAACAGGGCAGAGTAAG  R-CTAGTGTCCTTGGTGGTAGTCAT | *TaqI* | This study |
| BAC_119J05 | SL2.40ch11: 51.830 | F-AACTTACGGCACCTCAATTTTTC  R-GTGCCCCCTATGCAAGTAATTC | *None* | Ji et al. (2009) |
| T0302 | SL2.40ch11: 51.878 | F-TGGCTCATCCTGAAGCTGATAGCGC  R-TGATKTGATGTTCTCWTCTCTMGCCTG | *None* | Ji et al. (2009) |
| T0302-1 | SL2.40ch11: 51.878 | F-TGGCTCATCCTGAAGCTGAT  R-TGGAAAGGGATCGAAGAGAA | *None* | This study |
| TG105A | SL2.40ch11: 52.07 | F-ACATTTGGACAAATAGCAGAAGTC  R-TGAGAGCAGACAGCAGGCATCATC | *HpyCH4IV* | Ji et al. (2007b) |
| TG26 | SL2.40ch11: 52.53 | F-GTCGGTAACAGTTCTATGTTGCGG  R-TATTTGGTTCAGTCGTGGAGCC | *HinfI* | Ji et al. (2009a) |
| TG393 | SL2.40ch11: 53.25 | F-TGGATTTGATTAGCCGAAGG  R-CCAAGAATCCCAGAAGGAGA | *DpnII* | SGN |

**^a^** From Tomato WGS Chromosomes SL2.40 database at http://solgenomics.net/.

^b^ K= T/G; W = T/A; M = C/A.

^c^ SGN = Sol Genomics Network (http://www.sgn.cornell.edu).
