# Supplementary material for: Fine mapping of the tomato yellow leaf curl virus resistance gene Ty-2 on chromosome 11 of tomato
Source: Mol Breed. 2014 Mar 28;34(2):749–60. doi: 10.1007/s11032-014-0072-9 (PMC4092234; doi:10.1007/s11032-014-0072-9)
Supplement: Supplementary file 2 — Supplementary material 2 (DOCX 17 kb) [file 11032_2014_72_MOESM2_ESM.docx]

**Table S2.** Candidate genes in the *Ty-2* region. List shows predicted genes in the 300kb *Ty-2* region (based on the tomato genome sequence, Sol Genomics Network, SGN, www.solgenomics.net); 14 genes silenced and assessed for functionality so far are shown in bold

| Gene number | SGN gene name | Annotation | Expression^a^ | | | VIGS^b^ | |
| --- | --- | --- | --- | --- | --- | --- | --- |
| 1 | **Solyc11g069620.1** | **CC-NBS-LRR, resistance protein** | nd | | | **no phenotype** | |
| 2 | Solyc11g069630.1 | Receptor-like protein kinase At5g59670 | nd | | | | |
| 3 | Solyc11g069640.1 | Carbonic anhydrase family protein | nd | | | | |
| 4 | Solyc11g069650.1 | Unknown protein | nd | | | | |
| 5 | Solyc11g069660.1 | CC-NBS-LRR, resistance protein | down | | | | |
| 6 | **Solyc11g069670.1** | **Disease resistance protein R3a-like protein (fragment)** | nd | | | **no phenotype** | |
| 7 | Solyc11g069680.1 | Acyltransferase-like protein | nd | | | | |
| 8 | Solyc11g069690.1 | Protein disulfideisomerase | nd | | | | |
| 9 | **Solyc11g069700.1** | **Elongation factor 1-alpha** | down** | | **death** | | |
| 10 | Solyc11g069710.1 | ABC transporter G family member 3 | similar | | | | |
| 11 | **Solyc11g069720.1** | **26S protease regulatory subunit 6B homolog** | similar | | | | **no phenotype** |
| 12 | **Solyc11g069730.1** | **Unknown Protein** | similar | | | | **yellow leaves** |
| 13 | Solyc11g069740.1 | Nitrate transporter | ns | | | | |
| 14 | Solyc11g069750.1 | Nitrate transporter | ns | | | | |
| 15 | Solyc11g069760.1 | High affinity nitrate transporter protein | ns | | | | |
| 16 | **Solyc11g069770.1** | **Transcription factor MADS-box** | up* | | | **smaller yellowish plants** | |
| 17 | Solyc11g069780.1 | 2-phosphoglycerate kinase | up | | | | |
| 18 | **Solyc11g069790.1** | **Chaperonin** | similar | **no phenotype** | | | |
| 19 | **Solyc11g069800.1** | **Cytochrome P450** | ns **no phenotype** | | | | |
| 20 | **Solyc11g069810.1** | **OTU domain containing protein** | similar | | | | **no phenotype** |
| 21 | Solyc11g069820.1 | ABC transporter G family member 28 | down | | | | |
| 22 | **Solyc11g069830.1** | **Arsenite ATPase transporter (Eurofung)** | down **no phenotype** | | | | |
| 23 | **Solyc11g069840.1** | **Os03g0859900 protein** | similar | | | **no phenotype** | |
| 24 | **Solyc11g069850.1** | **Telomere repeat-binding protein 4** | similar | | | **no phenotype** | |
| 25 | Solyc11g069860.1 | Glutaredoxin | down | | | | |
| 26 | Solyc11g069870.1 | Ripening-related protein 3 | ns | | | | |
| 27 | Solyc11g069880.1 | Ripening-related protein 3 | down | | | | |
| 28 | Solyc11g069890.1 | BEL1-like homeodomain protein 8 | similar | | | | |
| 29 | Solyc11g069900.1 | Unknown Protein | similar | | | | |
| 30 | **Solyc11g069910.1** | **DNA-directed RNA polymerase II subunit J** | down* | **stunting, curling** | | | |
| 31 | Solyc11g069920.1 | Nbs, resistance protein fragment | similar | | | | |
| 32 | **Solyc11g069930.1** | **Disease resistance protein R3a-like protein** | down* | **no phenotype** | | | |
| 33 | Solyc11g069940.1 | Glutaredoxin | nd | | | | |
| 34 | Solyc11g069950.1 | Cell division protease ftsH homolog | nd | | | | |
| 35 | Solyc11g069960.1 | Receptor like kinase, RLK | nd | | | | |

^a^ Relative expression of genes on the resistant *Ty-2* line vs. susceptible (cv. Moneymaker) genotype. *indicates statistically differential up- or down-regulation (*=P<0.05, **=P<0.01); ns, not signal detected via RT-PCR; nd, not determined.

^b^VIGS: altered phenotype observed upon VIGS (virus-induced gene silencing).
