# Supplementary material for: Fine mapping of the tomato yellow leaf curl virus resistance gene Ty-2 on chromosome 11 of tomato
Source: Mol Breed. 2014 Mar 28;34(2):749–60. doi: 10.1007/s11032-014-0072-9 (PMC4092234; doi:10.1007/s11032-014-0072-9)
Supplement: Supplementary file 3 — Supplementary material 3 (DOCX 16 kb) [file 11032_2014_72_MOESM3_ESM.docx]

**Table S3.** Primers used for expression level analysis of candidate genes (RT-PCR)

| Gene number^a^ | Primer name | Sequence (5’-3’) | Target gene (SGN gene name) |
| --- | --- | --- | --- |
| 1 | rt620-F  rt620-R | TGATAGAAGGGAAGCCGTGAA  GTTTGAGAATCGTAAGCAGAACTACC | Solyc11g069620.1 |
| 5 | rt660-F  rt660-R | TGTGGAACAAGGTGGGCTTC  TGAGGTGGGAAAGATGTAGTGAATG | Solyc11g069660.1 |
| 9 | rt700-F  rt700-R | AGTATGCCTGGGTTCTTGAC  TGATGAAATCCCTATGACCA | Solyc11g069700.1 |
| 10 | rt710-F  rt710-R | GAAGTCAGAGTGGATGAAGGAGGTG  TAAAGGTGGAGATGGCAACGAAC | Solyc11g069710.1 |
| 11 | rt720-F  rt720-R | GTTAGAGGAGGGATGAGAAGTGTGG  AAGATTTCAGTGTCTGGAGCAACAA | Solyc11g069720.1 |
| 13 | rt740-F  rt740-R | AAATGGATGTTGGTAATGCTGGAGT  GTCACAAATCGCGCCCATAG | Solyc11g069740.1 |
| 14 | rt750-F  rt750-R | GGATGAGAGGAAGGTTATGGGTCTT  ATTGAAACTATGGCGATGGGTAATG | Solyc11g069750.1 |
| 15 | rt760-F  rt760-R | GAGAGGCAGACTATGGACATTATGGA  ATAGCCAGAGGAAGTGTGGTAGCC | Solyc11g069760.1 |
| 16 | rt770-F  rt770-R | GATGACACTGGCTCCCTCAG  TCTTCAAACTCTTCTTCAATCTCCA | Solyc11g069770.1 |
| 17 | rt780-F  rt780-R | TCTGAAGCGAAAGCGAAGAAA  TCTGGAACTAATCAAATCAACAGCA | Solyc11g069780.1 |
| 18 | rt790-F  rt790-R | CTCTGCTGGAAATGATGAAAGC  AGTTGTCTCTAAGGAGGAGGATGACT | Solyc11g069790.1 |
| 19 | rt800-F  rt800-R | GCTCGGTCGTTATTTGGAGTT  AGGTAATCGGAAAGTATGGAGGAG | Solyc11g069800.1 |
| 20 | rt810-F  rt810-R | CCTAGAATCAATGGAGAAATACCATCAA  ATCTGACAAAGCACGAAACT | Solyc11g069810.1 |
| 21 | rt820-F  rt820-R | ATGTTGGGCTGGAAATGGTTATG  GCTTCACGACGAGTTGCTCTG | Solyc11g069820.1 |
| 22 | rt830-F  rt830-R | AGGAGGGAAGGGAGGTGTGG  TGAAACAACAAGAGTCGGATGACC | Solyc11g069830.1 |
| 23 | rt840-F  rt840-R | CAGAGAACCAAGAAACCCGACAC  GTTGGAATTGGACTTTGCATGATTT | Solyc11g069840.1 |
| 24 | rt850-F  rt850-R | GAGACCCGAGGAACTAACCAAGACA  GCTCCATCTGCCAACACCATACTT | Solyc11g069850.1 |
| 25 | rt860-F  rt860-R | TTCTACATCGTTATCCTCCATACCC  ATGAATCTCCTCCTCCAGCAAC | Solyc11g069860.1 |
| 26 | rt870-F  rt870-R | ACAACTTTCATCTCTTCATCTTACTCCA  ACTTCCTCCTCCTCCACAAATACTG | Solyc11g069870.1 |
| 27 | rt880-F  rt880-R | CCCTCCTTCTGTCCCTTCTCC  GGTTAGTTGAGCGGGCGTTG | Solyc11g069880.1 |
| 28 | rt890-F  rt890-R | GAAACCGTGGCTGGTCTTAGTG  ATCTGTGATGGCGTTCCTCAGT | Solyc11g069890.1 |
| 29 | rt900-F  rt900-R | GACAACGACTTCTAGCTTTGCTACG  GCCCTCGTTCCAATAGGGTTT | Solyc11g069900.1 |
| 30 | rt910-F  rt910-R | AAGAGAGGACCATACAATCGGGAAC  CATAGGTGAGGACTGGCTTGTTGTT | Solyc11g069910.1 |
| 31 | rt920-F  rt920-R | GGAGAGTGTTGCTCAGACGATG  TTCAAGTGCTGGATGATTCCTATTT | Solyc11g069920.1 |
| 32 | rt930-F  rt930-R | CGGAAGCGTAAGAATGATGTTGAG  TGATTTGATGCCTGCTTATTCTCTG | Solyc11g069930.1 |

**^a^** The same as the gene number in Table S2.
