# Supplementary material for: Fine mapping of the tomato yellow leaf curl virus resistance gene Ty-2 on chromosome 11 of tomato
Source: Mol Breed. 2014 Mar 28;34(2):749–60. doi: 10.1007/s11032-014-0072-9 (PMC4092234; doi:10.1007/s11032-014-0072-9)
Supplement: Supplementary file 4 — Supplementary material 4 (DOCX 16 kb) [file 11032_2014_72_MOESM4_ESM.docx]

**Table S4.** Sequences of primers used for generation of VIGS constructs

| Gene number^a^ | | Primer name | Sequence (5’-3’) | Target gene (SGN gene name/Annotation) |
| --- | --- | --- | --- | --- |
| 1 | VG620-F  VG620-R | | CACCTCGGAAGCGTAAGAATGATGT  TCTACAGCATGTCGAAGCTCA | Solyc11g069620.1  CC-NBS-LRR, resistance protein |
| 6 | VG670-F  VG670-R | | CACCTCGGAAGCGTAAGAATGATGT  TCTACAGCATGTCGAAGCTCA | Solyc11g069670.1  Disease resistance protein R3a-like protein (fragment) |
| 9 | VG700-F  VG700-R | | CACCGGGTCCAACCCTTCTTGAGG  AGGCTCCTTCTCGAGTTCCT | Solyc11g069700.1  Elongation factor 1-alpha |
| 11 | VG720-F  VG720-R | | CACCGCACCAGTGTCCAGATGTCA  CTTTCCAGAACCAGGGGGTC | Solyc11g069720.1  26S protease regulatory subunit 6B homolog |
| 12 | VG730-F  VG730-R | | CACCACGAGTATTCGCGAGTTATCCA  TGTGAACATCCAATTGAGCCC | Solyc11g069730.1  Unknown Protein |
| 16 | VG770-F  VG770-R | | CACCCGCAGAAGAATCCGCATTGA  CCATTAGAGCCAACACCCCC | Solyc11g069770.1  Transcription factor MADS-box |
| 18 | VG790-F  VG790-R | | CACCACAAGGGTGCAAAGAACCGA  TGTCGTCACGGCCTTTAACA | Solyc11g069790.1  Chaperonin |
| 19 | VG800-F  VG800-R | | CACCGCAGTGATCAAGCTGCGTTC  GGATCTCCACCCCTGCTTTC | Solyc11g069800.1  Cytochrome P450 |
| 20 | VG810-F  VG810-R | | CACCGCTTTCAGTCGCTGAACCAC  GGCCATGGGAACATATCCGT | Solyc11g069810.1  OTU domain containing protein |
| 22 | VG830-F  VG830-R | | CACCTGGTTAGTGGGACACAACGG  CTGCGTGGAACTCTTCCCTT | Solyc11g069830.1  Arsenite ATPase transporter (Eurofung) |
| 23 | VG840-F  VG840-R | | CACCGCATCAACATCTATGGAGCCC  TGCTGGAGAAGAACGTGTGC | Solyc11g069840.1  Os03g0859900 protein |
| 24 | VG850-F  VG850-R | | CACCTTCGCGAGGAGGATTTGGTC  CAGCTGGACTACGATGCACA | Solyc11g069850.1  Telomererepeat-binding protein 4 |
| 30 | VG910-F  VG910-R | | CACCTTCGTTGTTCCAGAAGGCGT  AGGTGAGGACTGGCTTGTTG | Solyc11g069910.1  DNA-directed RNA polymerase II subunit J |
| 32 | VG930-F  VG930-R | | CACCTCGGAAGCGTAAGAATGATGT  TCTACAGCATGTCGAAGCTCA | Solyc11g069930.1  Disease resistance protein R3a-like protein |

**^a^** The same as the gene number in Table S2.
