# Supplementary figures and images for: Fine mapping of the tomato yellow leaf curl virus resistance gene Ty-2 on chromosome 11 of tomato
Source: Mol Breed. 2014 Mar 28;34(2):749–60. doi: 10.1007/s11032-014-0072-9 (PMC4092234; doi:10.1007/s11032-014-0072-9)

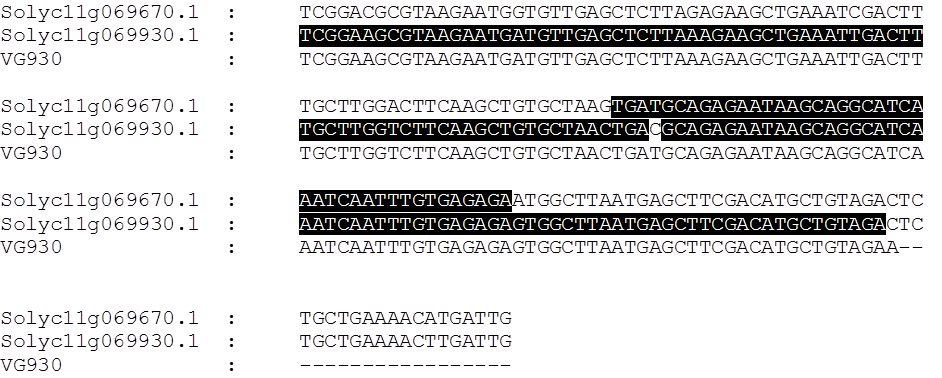

Supplement: Supplementary file 5 — Supplementary material 5 Figure S1. Target regions for silencing the R3a homologs in tomato chromosome 11. Nucleotide sequence alignments of predicted R3a homologs in the Ty-2 region are shown: Disease resistance protein R3a-like fragment (Solyc11g069670.1), Disease resistance protein R3a-like protein (Solyc11g069930.1), and TRV-based VIGS construct VG930. Regions highlighted in black represent sequences targeted for VIGS for each predicted gene. cDNA sequences were obtained from SGN public database (TIFF 68 kb) [file 11032_2014_72_MOESM5_ESM.tif]
